# Retinal and Choroidal Vascular Changes in Eyes with Pseudoexfoliation Syndrome: A Comparative Study Using Optical Coherence Tomography Angiography

**DOI:** 10.4274/balkanmedj.galenos.2019.2019.5.5

**Published:** 2019-12-20

**Authors:** Esat Çınar, Berna Yüce, Fatih Aslan

**Affiliations:** 1 Clinic of Ophthalmology, Ekol Hospital, İzmir, Turkey; 2 Clinic of Ophthalmology, İzmir University of Health Sciences, Tepecik Training and Research Hospital, İzmir, Turkey; 3 Clinic of Ophthalmology, Alaaddin Keykubat University Training and Research Hospital, Alanya, Antalya, Turkey

**Keywords:** Choroid, optical coherence tomography angiography, pseudoexfoliation, retina, vascular structure

## Abstract

**Background:**

Optical coherence tomography angiography allows a detailed evaluation of retinal and choroidal microvascular structures without the need for a contrast agent. Pseudoexfoliation syndrome is a condition that leads to anatomical and functional losses due to accumulation of degraded abnormal fibrillar material in the intraocular and extraocular tissues. Histopathological studies have shown that the accumulation of Pseudoexfoliation syndrome material in the vascular structures may play a role in different ocular pathologies such as retinal vein occlusion, iris hypoperfusion, anterior segment hypoxia, retinal arterial occlusion, and neovascular glaucoma.

**Aims:**

To evaluate and compare flow and vascular density in the retina and choroid in eyes with Pseudoexfoliation syndrome, fellow eyes without Pseudoexfoliation syndrome, and healthy eyes using optical coherence tomography angiography.

**Study Design:**

Case control study.

**Methods:**

The study included 35 eyes with Pseudoexfoliation syndrome of 35 Pseudoexfoliation syndrome patients, 32 fellow eyes without Pseudoexfoliation syndrome of 32 unilateral Pseudoexfoliation syndrome patients, and 35 eyes of healthy control subjects. Flow area and vascular density in the superficial capillary plexus and deep capillary plexus were measured by optical coherence tomography angiography as three separate parameters: total, parafoveal, and foveal. Choroidal thickness and foveal avascular zone area were measured for each patient.

**Results:**

There were significant differences between the Pseudoexfoliation syndrome eyes and control eyes in total, parafoveal, and foveal flow and vascular density in the superficial capillary plexus (p<0.05 for all), while there were no significant differences between these groups in any of the flow or vascular density values in the deep capillary plexus (p>0.05). None of the superficial capillary plexus and deep capillary plexus flow and vascular density values showed significant differences between Pseudoexfoliation syndrome eyes and non-Pseudoexfoliation syndrome fellow eyes or between the non-Pseudoexfoliation syndrome fellow eyes and control eyes (p>0.05). Choroidal thickness was significantly lower in Pseudoexfoliation syndrome eyes compared to control eyes. Foveal avascular zone area was significantly enlarged in Pseudoexfoliation syndrome eyes compared to control eyes in both the superficial and deep layers (p<0.05).

**Conclusion:**

Pseudoexfoliation syndrome eyes exhibit significant damage to the retinal and choroidal vascular structures.

Although retinal veins can be directly visualized, measuring retinal flow noninvasively was not possible before the development of Doppler techniques (1). Optical coherence tomography angiography (OCTA) is a new imaging modality that measures the red blood cell velocity. OCTA allows a detailed evaluation of retinal and choroidal microvascular structures without the need for a contrast agent. To date, OCTA has been used in various retinal vascular pathologies such as diabetic retinopathy, age-related macular degeneration, retinal vein occlusion, macular telangiectasia, and choroidal neovascularization (2).

Pseudoexfoliation syndrome (PEX) is a condition that leads to anatomical and functional losses due to accumulation of degraded abnormal fibrillar material in the extracellular spaces of intraocular and extraocular tissues. Although it is frequently associated with glaucoma, the accumulation of PEX material in the retinal vasculature is known to cause vasculopathy as well as disrupt the perfusion by damaging the walls of the posterior ciliary arteries and vortex vein (3,4,5). Histopathological studies have shown that the accumulation of PEX material in the vascular structures may play a role in different pathologies such as retinal vein occlusion, iris hypoperfusion, anterior segment hypoxia, retinal arterial occlusion, and neovascular glaucoma (6,7,8).

In the present study, we sought to identify PEX-related changes in the retinal vasculature by evaluating retinal and choroidal flow and vessel density using OCTA in eyes with biomicroscopically detected PEX material and comparing them with the fellow eyes not involved with PEX of these patients and a healthy control group.

## MATERIALS AND METHODS

This cross-sectional study included 35 patients (19 females, 16 males; mean age 69.8±6.9 years) diagnosed with PEX and 35 healthy individuals (18 females, 17 males; mean age 68±5.5 years) with no known ocular disease. Three of the 35 PEX patients had bilateral involvement, while the other 32 had unilateral involvement. Therefore, the eyes were divided into 3 groups: eyes with PEX (35 eyes), fellow eyes without PEX of the PEX patients with unilateral involvement (32 eyes), and healthy control eyes (35 eyes). In the PEX patients with bilateral involvement and in the healthy subjects, one eye was randomly selected for analysis using random number tables (odd number=left eye; even number=right eye).

The study was conducted in accordance with the Declaration of Helsinki and an informed consent was obtained from each of the participants. The study was approved by the Ethics Committee of Alaaddin Keykubat University (ethics committee no: 5-9/2018).

All patients had visual acuity of 1.0 according to Snellen chart, and slit-lamp anterior segment and dilated fundus examinations were normal. To avoid affecting the OCTA measurements, all participants selected for the study had spherical refractive error values less than or equal to ±4.00 diopters and cylindrical values less than or equal to ±3 diopters. Exclusion criteria included history of ocular surgery, presence of systemic disease (diabetes mellitus, hypertension, etc.), use of systemic medication or eye drops other than artificial tears, and presence of corneal opacity or cataract higher than grade 1 in severity. Those with grade 1 cataract and those using artificial tear eye drops were included in the study.

All OCTA measurements and imaging procedures were performed with the pupil dilated, using an AngioRTVue XR (Optovue Inc., Freemount, CA) with version 2015.1.1.98 software, by a qualified technician trained at using the equipment.

### Optical coherence tomography angiography imaging

**Figure 1:**
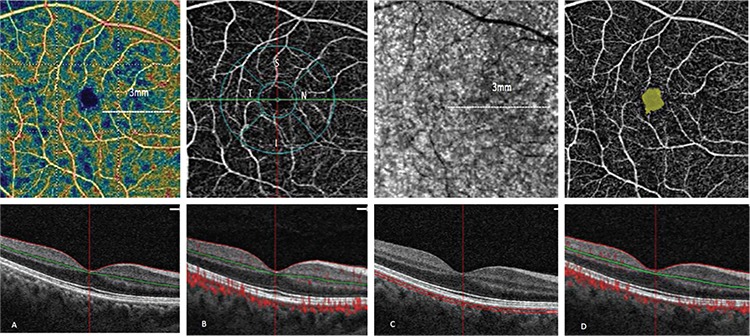
a-d. Macular perfusion parameters of a 6 mm×6 mm angiography scan size using optical coherence tomography angiography. (a) The flow area within a 3 mm radius is represented by yellow; (b) The vessel density of five areas of interest including the fovea (1 mm diameter) and temporal, inferior, nasal, and superior quadrants (1 mm annular ring); (c) the choroidal capillary flow area within a 3-mm radius is represented by yellow; (d) the foveal avascular area is automatically delineated using the included software and represented by yellow.

An area map of 6×6 mm was used in all images. Optovue Angio-Vue system technology allows for a quantitative analysis. The inner boundary for superficial capillary plexus was assumed as 2.6 µm below the internal limiting membrane and the outer boundary as 15.6 µm below the inner plexiform layer. The inner boundary for deep capillary plexus was assumed as 15.6 µm and the outer boundary as 70.2 µm below the inner plexiform layer (Figure 1a, b). Thus, the 15.6-70.2 µm area from the retinal surface was considered as deep capillary plexus and its flow was also measured. For each patient, the area of the central macula with no detected vascular structures was automatically measured and recorded as the foveal avascular zone (Figure 1d). The deep capillary plexus starts from the internal limiting membrane and extends to the retinal pigment epithelium. For measurements of the choroidal layer, the retinal pigment epithelium was used as a reference point and the areas under retinal pigment epithelium were measured (Figure 1c). Flow and vessel density in the superficial capillary plexus and deep capillary plexus were evaluated as three different parameters: total, parafoveal, and foveal. A total of 5 choroidal thickness measurements were obtained by manually measuring at the fovea (subfoveal) and at distances of 500 and 1,000 μm nasal and temporal to the fovea. Retinal thickness was also measured at the fovea.

To avoid diurnal fluctuations in choroidal thickness, measurements were performed in the same time interval (10:00 a.m. - 12:00 p.m.) for all the participants. Only those with good image quality (>72 of 100) were included in the study.

### Statistical analysis

We calculated the number of patients needed to obtain the desired effect size. Considering the results of a previous study (9) that detected a significant difference in mean peripapillary capillary density between pseudoexfoliative glaucoma and primary open-angle glaucoma using Angiovue OCTA and assuming an α error of 0.05% and power of 85%, the required sample size was calculated as 32 subjects per group.

In this study, one eye per subject was enrolled. Continuous variables were described as mean and standard deviation. The distribution of numerical data was tested for normality using the one-sample Kolmogorov-Smirnov test. Significant differences between continuous variables across the groups were tested with one-way analysis of variance with Bonferroni post hoc analysis. SPSS software (version 16.0 for Windows; SPSS Inc., Chicago, IL, USA) was used for all analyses. A p value <0.05 was considered statistically significant.

## RESULTS

There was no difference in age or sex ratio between the PEX patient group and control group (age, p=0.814; sex, p=0.943). Comparisons of the PEX patients’ affected eyes and unaffected fellow eyes and the healthy control eyes are shown in Table 1.

**Figure 2:**
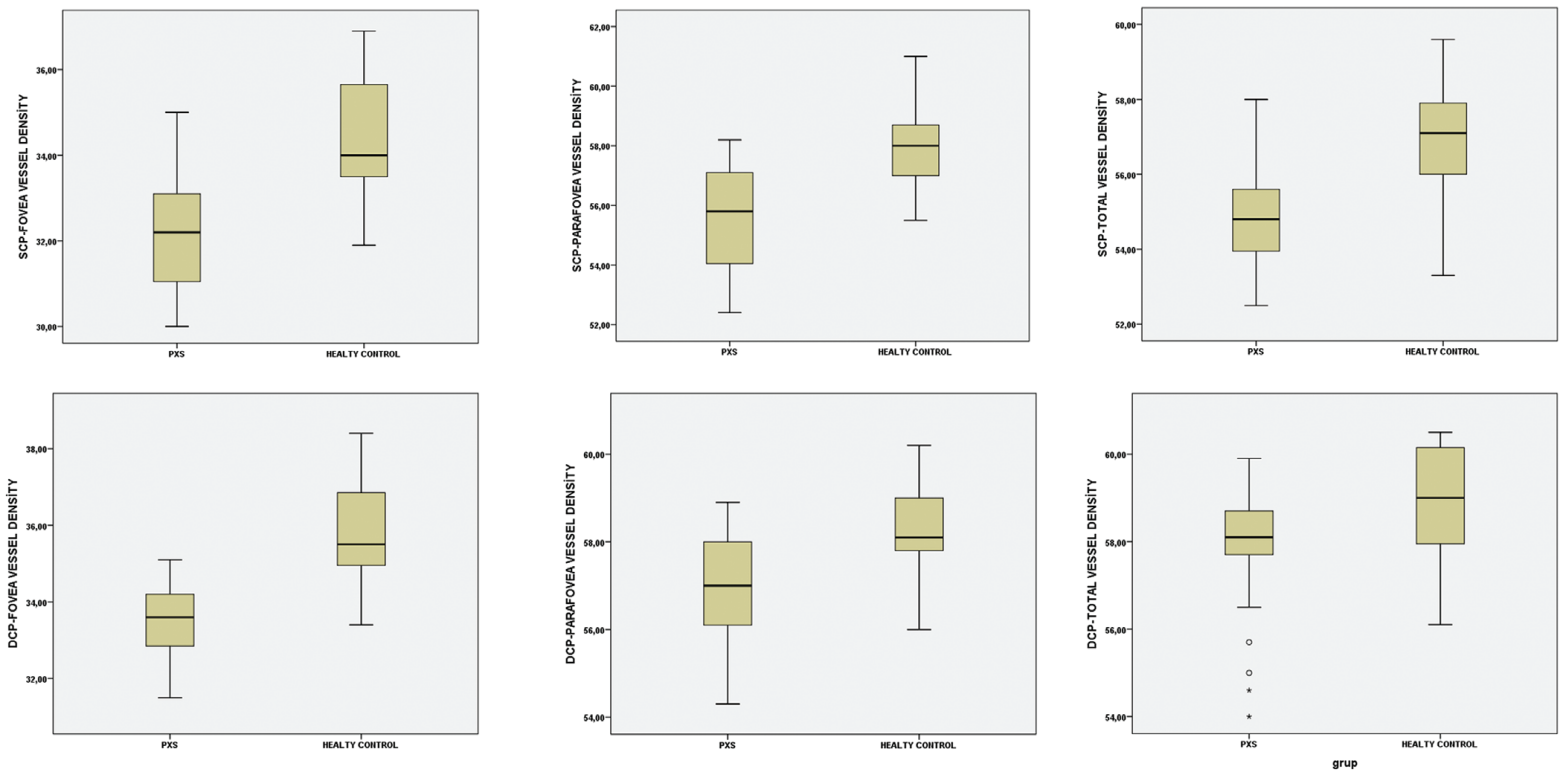
The box plot analysis representing the superficial and deep capillary plexus vessel density in the pseudoexfoliation syndrome patients and control subjects.

PEX eyes had significantly lower total, parafoveal, and foveal superficial capillary plexus flow compared to healthy control eyes (p<0.05, for all); however, there were no differences in these parameters when compared with the non-PEX fellow eyes (p>0.05, for all). Likewise, the total, parafoveal, and foveal vessel densities in the superficial capillary plexus were also significantly reduced in PEX eyes compared to those in healthy control eyes (p<0.05, for all), while no significant differences were detected compared to those in fellow non-PEX eyes (p>0.05, for all). Details are shown in Table 1. The box plot analysis representing the superficial capillary plexus and deep capillary plexus vessel densities in the PEX patients and control subjects are shown in Figure 2.

The PEX eyes showed significantly lower flow in the total, parafoveal, and foveal area in deep capillary plexus when compared to control eyes (p<0.05, for all); however, there was no significant difference in flow compared to that in the fellow non-PEX eyes in any of the areas (p>0.05, for all). In addition, vessel density in the total, parafoveal, and foveal areas in deep capillary plexus was significantly lower in PEX eyes compared to that in the healthy eyes (p<0.05, for all); whereas, no significant differences emerged between the PEX eyes and non-PEX fellow eyes (p>0.05, for all) Details are shown in Table 1.

PEX eyes exhibited significant foveal avascular zone enlargement in both the superficial capillary plexus and deep capillary plexus compared to the non-PEX fellow eyes and healthy control eyes (p<0.05, for all).

There were no significant differences between the groups in terms of subfoveal retinal thickness (p>0.05, for all). The subfoveal choroidal thickness did not differ statistically between the PEX and fellow eyes (p=0.413), but was significantly lower in the PEX eyes when compared with that in healthy control eyes (p=0.021).

## DISCUSSION

PEX is characterized by the accumulation of abnormal fibrillar material in the extracellular matrix of the ocular tissues, which has been shown to cause clinically observable pathologies such as glaucoma, zonular weakness, and iris atrophy, as well as vascular dysfunction due to its accumulation on vessel walls, which is not directly observable (4,5,6,7,8).

In the present study, OCTA measurements demonstrated significant decline in superficial retinal blood flow and vessel density in eyes with PEX compared to that in the healthy eyes. Although significant disruption in deep retinal flow and deep vessel density was not observed, enlargement of the foveal avascular zone in both superficial and deep retinal layers supports the existence of a PEX-related vascular pathology.

The role of PEX in the retinal vasculature has been debated for many years. A retrospective study evaluating 332 patients with branch retinal vein occlusion and 159 patients with central retinal vein occlusion revealed PEX material in 6% of the branch retinal vein occlusion patients and 6.9% of the central retinal vein occlusion patients, providing important evidence that PEX material creates a tendency for vascular thrombosis (6). Yüksel et al. (10) used color Doppler ultrasound to measure peak systolic and diastolic flow in the ophthalmic artery, central retinal artery, short posterior ciliary arteries, and temporal ciliary arteries of 14 eyes with pseudoexfoliative glaucoma, 14 PEX eyes without glaucoma, and 14 healthy eyes, and reported a significant reduction in blood flow in eyes with PEX regardless of the presence of glaucoma. Ocakoglu et al. (11) compared 22 eyes with unilateral PEX with the patients’ fellow eyes and 22 healthy eyes and found that both PEX and non-PEX fellow eyes had significantly disrupted flow in the optic nerve head and peripapillary area when compared with that in the healthy control eyes. Studies have not only demonstrated that PEX material is correlated with vascular dysfunction, but have also identified it as an independent risk factor (6). In a recent study comparing ophthalmic arterial hemodynamics and vascular resistance in eyes with PEX and healthy eyes, Kocaturk et al. (12) reported an increase in vascular resistance and disruption of hemodynamic parameters in PEX eyes. The results of our study corroborate previous studies demonstrating retinal blood flow abnormalities in patients with PEX compared with healthy individuals (6,10,11,12).

In a histological study conducted using electron microscopy, comparison of the posterior segment vascular structures in 120 eyes enucleated due to central retinal vein occlusion-related complications and 107 eyes enucleated due to melanoma, PEX material was detected in 10% of the central retinal vein occlusion eyes and 1.9% of the melanoma eyes, and the authors indicated that PEX may have a role in vascular pathologies (13). In another recent study, Karagiannis et al. (14) detected PEX material in 29.17% of eyes with central retinal vein occlusion and 8.5% of eyes with branch retinal vein occlusion, showing that these rates were higher than in healthy eyes. Moreover, they proposed that the high amount of PEX material in eyes with central retinal vein occlusion may be an independent risk factor in the etiology of central retinal vein occlusion (14).

Park and Yoo (15) compared peripapillary optic nerve vessel density and retinal nerve fiber layer thickness in 39 eyes with pseudoexfoliative glaucoma and 39 eyes with primary open-angle glaucoma. Their study showed that although peripapillary vessel density was significantly lower in patients with PEX, there was no significant difference between the two groups in terms of retinal nerve fiber layer thickness. The authors stated that in addition to glaucoma, PEX material may have precipitated an ischemic event in the peripapillary area due to the damage it caused, particularly in the endothelium of the small vessels. Kromer et al. (16) evaluated macular vessel density and flow in 30 individuals with primary open-angle glaucoma and 21 healthy individuals, using OCTA. Global and nasal vessel density were reduced in primary open-angle glaucoma patients compared to the healthy individuals, but no difference was observed in terms of flow. However, they did not perform an additional subgroup analysis between eyes with pseudoexfoliative glaucoma, eyes with other types of glaucoma, and healthy eyes. The results from Park, Kromer, and our study suggest that although having primary open-angle glaucoma alone causes a decrease in both macular and peripapillary vessel density, the presence of PEX material in addition to primary open-angle glaucoma also leads to flow disruption. This may be a result of PEX material inducing ischemia, as stated by Park. Our finding that the PEX material is an independent risk factor supports the study by Park and Yoo (15).

The superficial retinal vascular plexus is closely related to the ganglion cell layer and has a critical role in supplying it. Eltutar et al. (17) compared OCT findings in 45 eyes with PEX and 29 healthy non-PEX eyes and demonstrated that the ganglion cell layer and superficial retinal layer were significantly thinner in PEX eyes and that this thinning may be an early sign of pseudoexfoliative glaucoma in patients with PEX. The reduced flow and vessel density in the superficial retinal layers observed in our study are important findings that may explain the thinning of the ganglion cell layer reported by Eltutar et al. (17).

Another finding of our study was significant enlargement of the foveal avascular zone measured in both the superficial and deep layers in PEX patients compared with that in the healthy individuals. Freiberg et al. (18) compared foveal avascular zone area in the superficial and deep layers in 29 patients with diabetic retinopathy and 25 healthy eyes using OCTA and found that eyes with diabetic retinopathy had significant foveal avascular zone enlargement and irregularity in both the superficial and deep layers. The authors emphasized the importance of altered foveal avascular zone area in diseases of vascular etiology. Wons et al. (19) indicated that visual acuity was associated with foveal avascular zone diameter in patients with central retinal vein occlusion and branch retinal vein occlusion and that destruction of vascular structures in foveal avascular zone may be an important indicator. The foveal avascular zone enlargement shown in our study supports the correlation between foveal avascular zone and vascular dysfunction demonstrated by Freiberg and Wons. Assessing foveal avascular zone with OCTA may be useful for identifying PEX-associated vasculopathy or as an indicator of ocular tissue damage due to PEX, and further studies on this subject are needed.

Our comparison of PEX eyes, non-PEX fellow eyes, and healthy eyes revealed no significant differences in the deep capillary plexus in the outer plexiform layer. Although our data indicate that both flow and vessel density in the deep vascular plexus were affected by PEX, these differences did not reach a statistical significance. PEX is known to generally show unilateral involvement, although it can also appear in the fellow eye in later years (20). Histopathologic studies using electron microscopy have demonstrated that a certain amount of PEX material is present in the conjunctiva, iris, and especially the iris dilator muscles of eyes in which PEX involvement is not detected biomicroscopically (21). In addition, clinical studies have clearly demonstrated vascular dysfunction in fellow eyes with no apparent PEX material (10,11). Both clinical and histological studies have shown that although PEX material may appear biomicroscopically unilateral, it is actually present in the fellow eye at a microscopic level. We also observed lower retinochoroidal flow and vessel density in the non-PEX fellow eyes of PEX patients in the current study. Although the differences were not statistically significant, our findings support the possibility of undetected PEX material in apparently unaffected fellow eyes.

In this study, we observed no significant differences among the eyes in terms of retinal thickness at the fovea. However, there was a significant reduction in mean choroidal thickness in the PEX eyes compared with that in the healthy eyes. Demircan et al. (22) compared choroidal thickness in 43 eyes with pseudoexfoliative glaucoma, 45 PEX eyes without glaucoma, and 48 healthy eyes using enhanced depth imaging-OCT. They demonstrated significant choroidal thinning in eyes with PEX material, regardless of the presence of glaucoma, and stated that this may be due to ischemia in the choroidal vessels caused by the PEX material (22). Eroglu et al. (23) also demonstrated significant choroidal thinning in the eyes of patients with bilateral PEX compared to that in healthy eyes and eyes with unilateral PEX. In the same study, they showed that patients with unilateral PEX also had significant choroidal thinning compared to that in the healthy individuals, indicating that PEX material significantly affects choroidal thickness. Our findings of reduced choroidal thickness in eyes with PEX suggest that this may be due to changes in the choroidal blood flow resulting from existing vasculopathy, which both confirms the choroidal thinning observed in previous studies as well as sheds light on its possible causes. However, more data are needed on this subject.

Rebolleda et al. (9) compared peripapillary retinal nerve fiber layer thickness and peripapillary capillary density values obtained by two different OCTA devices in 20 eyes with pseudoexfoliative glaucoma, 20 eyes with primary open-angle glaucoma, and 20 healthy eyes. They reported that only Angiovue detected significantly lower capillary density in pseudoexfoliative glaucoma compared to that in primary open-angle glaucoma at similar levels of glaucoma damage, while both Angiovue and Angioplex demonstrated decreased capillary density in glaucoma eyes compared to in healthy eyes. Zengin et al. (24) evaluated the relationship between age-related macular degeneration and clinically unilateral PEX syndrome and showed that PEX was associated with a lower prevalence of wet age-related macular degeneration. As demonstrated in both glaucoma and age-related macular degeneration studies, PEX material may be involved in the etiopathogenesis of these conditions. Histopathologic studies are needed to clarify the causal relationship between PEX material and retinochoroidal diseases.

It has been shown that PEX patients are prone to thrombosis and vascular pathologies because they have higher blood homocysteine levels compared to healthy individuals, and the PEX biomarkers such as amyloid β peptide accumulate and lead to dysfunction in various tissues (25,26). It has also been suggested that increased levels of various local mediators of vasoconstriction such as endothelin-1 and decreased aqueous levels of strong mediators of vasodilation such as nitric oxide may play a role in vascular occlusion in eyes with PEX (27,28). In our study, both flow and vessel density were reduced. All these findings indicate that PEX patients have multiple risk factors, and the decrease in retinochoroidal flow may not be solely attributable to the PEX material, but intermediate mediators may also be involved.

Limitations of our study are that it included a small number of patients and was cross-sectional in design. Data from patients with PEX can be better demonstrated through longitudinal studies. In addition, there is a potential for human error in manual choroidal thickness measurement. Strengths of our study are the use of OCTA, which enables a rapid and noninvasive measurement of vascular flow, and the inclusion of a control group of healthy eyes for comparison.

In conclusion, we believe that the decrease in retinal and choroidal vessel density observed in our study may help elucidate the pathophysiology of glaucoma and other retinochoroidal diseases associated with PEX. Future studies investigating systemic (e.g., homocysteine, amyloid beta peptide) and local factors (e.g., endothelin, lysyl oxidase-like 1, nitric oxide) together with OCTA evaluation of retinochoroidal circulation may further advance our understanding of the pathophysiology of PEX.

## Figures and Tables

**Table 1:**
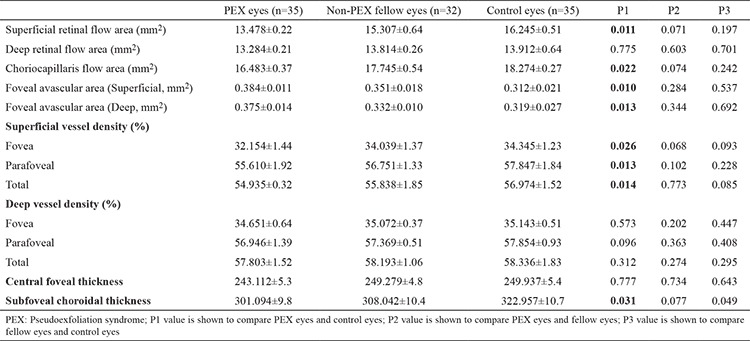
Comparisons of the PEX patients’ affected eyes and unaffected fellow eyes and the healthy control eyes
